# Comparison of the Conventional, Chemical, and Ultrasound Extraction of Crude Polysaccharides and Their Properties from *Lentinula edodes (Berk.) Pegler*

**DOI:** 10.3390/foods14142428

**Published:** 2025-07-09

**Authors:** Nannapat Phosarith, Thanyaporn Siriwoharn, Wachira Jirarattanarangsri

**Affiliations:** Division of Food Science and Technology, Faculty of Agro-Industry, Chiang Mai University, Chiang Mai 50100, Thailand; nannapat_psr@hotmail.com

**Keywords:** *Lentinula edodes*, β-glucan, extraction methods, enzyme inhibition, functional properties

## Abstract

This study aimed to compare the efficiency of four extraction methods, hot water (HW), hot alkaline (HA), ultrasound-assisted water (UW), and ultrasound-assisted alkaline (UA), for extracting crude β-glucan from *Lentinula edodes*, focusing on yield, functionality, and antidiabetic potential. The response surface methodology was used to optimize extraction conditions. Among all methods, UW yielded the highest β-glucan content (34.51 ± 0.82 g/100 g dry extract), indicating enhanced extraction efficiency through acoustic cavitation. However, HW demonstrated the most preserved structural integrity, exhibiting superior and consistent swelling power across all tested pH conditions, which indicated an excellent water-holding capacity. The ability of HA to scavenge antioxidants was significantly higher than that of other methods, likely due to the enhanced release of phenolic residues under alkaline conditions. UA showed the most potent inhibition against α-amylase (IC_50_ = 1.46 mg/mL) and α-glucosidase (IC_50_ = 1.21 mg/mL), demonstrating the potential for type 2 diabetes management. These results highlight that while UW is optimal for yield, HW preserves functional integrity, HA enhances antioxidant properties, and UA is promising for enzyme inhibition. The findings provide insights into tailoring extraction strategies for targeted functional or nutraceutical applications.

## 1. Introduction

Metabolic syndrome and obesity have significant roles in the development of noncommunicable diseases (NCDs), such as type 2 diabetes (T2D), cardiovascular disease, and lipid metabolism disorders [1]. T2D, which accounts for about 96% of all diabetes cases, is a serious global health issue defined by chronic hyperglycemia and insulin resistance. Poor dietary habits, particularly the excessive intake of high-sugar foods, contribute to the rapid rise in blood glucose levels, prompting excessive insulin production. Over time, this leads to insulin resistance and eventual β-cell dysfunction, leading to chronic hyperglycemia. Global diabetes healthcare expenses are expected to exceed USD 1054 billion by 2045, underscoring the urgent need for improved preventative and treatment strategies [2].

The consumption of high-fiber foods is increasingly recognized as an important strategy for maintaining health and preventing disease [3]. *Lentinula edodes (Berk.) Pegler*, also known as shiitake mushroom, is widely cultivated across Asia—including the northern regions of Thailand [4]. Dietary fiber, comprising both soluble and insoluble types, constitutes an important nutritional component of *L. edodes*. Among these, β-glucan (BG), a water-soluble polysaccharide primarily located in the fungal cell walls, has garnered significant attention due to its various health-promoting properties [5]. β-glucans extracted from mushrooms are composed of glucose residues primarily linked by β-(1→3) glycosidic bonds with β-(1→6) branches [6]. These compounds have specific immunomodulatory properties and a wide range of bioactivities, including antioxidants, anti-inflammatory, cholesterol-lowering, and blood-glucose-regulating actions [7]. While several studies have explored polysaccharide extraction from *L. edodes*, most have focused on yield or general antioxidant activity without a systematic comparison of how extraction methods influence both structural characteristics and targeted bioactivities such as α-amylase and α-glucosidase inhibition. The method used for β-glucan extraction highly depends on the structural composition of fungal cell walls [8]. Traditional extraction methods often involve hot water extraction or alkaline-/acid-based extraction. These procedures are relatively straightforward, typically involving grinding the mushroom samples, boiling them in hot water or chemical solvents, followed by filtration and precipitation to isolate β-glucan. While these methods are cost-effective and convenient, they are limited by the requirement for extended extraction times and high temperatures [9]. In recent years, ultrasonic-assisted extraction (UAE) has emerged as a promising technique for extracting bioactive compounds, including β-glucans. This method utilizes high-frequency ultrasonic waves that generate cavitation and acoustic vibrations in the solvent, disrupting the integrity of the fungal cell walls and enhancing the contact between the solvent and target compounds. Consequently, the UAE significantly improves the extraction efficiency [10]. In this study, although the UAE conditions matched conventional methods in time and solvent volume, prolonged ultrasonication was intentionally applied to explore its effects on polysaccharide yield and bioactivity, allowing for a controlled assessment of structural and functional modifications rather than focusing only on process efficiency. Few studies have conducted a comprehensive side-by-side evaluation of hot water-, alkaline-, and ultrasound-assisted extraction methods, assessing not only β-glucan yield and molecular characteristics but also their impact on antioxidant capacity and enzyme inhibition relevant to glycemic control. Moreover, the inclusion of β-glucan recovery efficiency and bioactivity-to-yield ratios adds mechanistic insight that enhances the translational value of this research.

The primary objective of this study was to investigate and compare the efficiency of different extraction techniques for β-glucan from *L. edodes*, with the aim of identifying the optimal conditions for each method to maximize both yield and quality. Particular attention was given to the preservation of the functional structure of crude polysaccharides and their bioactivity related to the inhibition of carbohydrate-hydrolyzing enzymes, which are associated with type 2 diabetes management. The outcomes of this research are expected to determine the most effective extraction approach that maintains the structural integrity of β-glucan and enhances its potential as a bioactive compound. This could support its future application in the development of health-promoting agents, particularly in the nutraceutical and functional food industries.

## 2. Materials and Methods

### 2.1. Materials and Chemicals

Dried *L. edodes* mushrooms were purchased from local supermarkets in Chiang Mai, Thailand. The dried mushrooms were ground using a hammer mill and passed through a mesh to obtain powder with a particle size of less than 0.5 mm. The powdered samples were stored at 4 °C in sealed containers for use.

Monobasic sodium phosphate (NaH_2_PO_4_); dibasic sodium phosphate (Na_2_HPO_4_); 1, 1-Diphenyl-2-picrylhydrazyl (DPPH); 2,2′-Azino-bis (3-ethylbenzothiazoline-6-sulfonic acid; TPTZ (2,4,6-tripyridyl-s-triazine); and sodium carbonate (Na_2_CO_3_) were purchased from Sigma–Aldrich, St. Louis, MO, USA. Ethanol, methanol, sodium hydroxide, glacial acetic acid, and hydrochloric acid were purchased from RCI Labscan Ltd., Bangkok, Thailand. Folin–Ciocalteu reagent and Potassium bromide (KBr) were purchased from Merck, Darmstadt, Germany. Acarbose and 4-Nitrophenyl-α-D-glucopyranoside were purchased from Macklin, Shanghai, China α-amylase (Porcine pancreatic: EC 3.2.1.1), α-glucosidase (Bacillus stearothermophilus: EC 3.2.1.20), and β-Glucan Assay Kit (Yeast and Mushroom) were obtained from Megazyme International (Bray, County Wicklow, Ireland).

### 2.2. Hot Water Extraction (HW)

The extraction was performed as described earlier with some modifications [11]. A total of 10 g of *L. edodes* powder was mixed with distilled water at different solid-to-liquid ratios (1:10, 1:20, 1:30 *w*/*v*). The mixtures were subjected to extraction at various temperatures (70, 80, and 90 °C) for different times (30, 60, and 90 min). After extraction, samples were centrifuged at 8000 rpm for 10 min at 4 °C to separate the supernatant from the residue. Each supernatant was mixed with 95% ethanol (1:2 *v*/*v*) and incubated overnight at 4 °C to facilitate precipitation. The mixture was then centrifuged again under the same conditions. The resulting precipitate was collected, dried in a vacuum oven at 65 °C, and stored at −18 °C until further analysis.

### 2.3. Hot Alkaline Extraction (HA)

The extraction was performed as described earlier with some modifications [11]. A total of 10 g of *L. edodes* powder was mixed with 0.5 M NaOH (pH 12) at varying solid-to-liquid ratios (1:10, 1:20, and 1:30 *w*/*v*). The suspensions were heated at different temperatures (70, 80, and 90 °C) for different times (30, 60, and 90 min). After extraction, samples were centrifuged at 8000 rpm for 10 min at 4 °C. The supernatant was collected, neutralized to pH 7, and precipitated by adding 95% ethanol (1:20, *v*/*v*), followed by incubation at 4 °C overnight. The resulting precipitate was recovered by centrifugation (8000 rpm, 10 min, 4 °C), dried in a vacuum oven at 65 °C, and stored at −18 °C.

### 2.4. Ultrasound-Assisted Water Extraction (UW)

UW was performed with modifications based on a previously described method [12,13,14]. A total of 10 g of dried *L. edodes* powder was suspended in distilled water at ratios of 1:10, 1:20, and 1:30 (*w*/*v*). The mixtures were treated using an ultrasonic processor equipped with a 25 mm probe (20 kHz frequency, 750 W maximum power, Model: SONIC 3, SONIC Corporation, Newtown, CT, USA) at varying amplitudes (40%, 50%, and 60%) for different durations (30, 60, and 90 min). The temperature did not exceed 60 °C, using an external water bath for temperature control, and the ultrasonic operation was applied in an intermittent mode (4 s on, 3 s off). After treatment, samples were centrifuged at 8000 rpm for 10 min at 4 °C. The supernatant was mixed with 95% ethanol (1:2, *v*/*v*) and incubated at 4 °C overnight. After centrifugation under the same conditions, the crude extract precipitate was dried at 65 °C in a vacuum oven and stored at −18 °C.

### 2.5. Ultrasound-Assisted Alkaline Extraction (UA)

UA extraction was carried out similarly to the UW method [12,13,14], except that the extraction medium was 0.5 M NaOH (pH 12). Dried *L. edodes* powder (10 g) was mixed with the alkaline solution at different solid-to-liquid ratios (1:10, 1:20, and 1:30 *w*/*v*). The mixtures were treated with an ultrasonic processor (25 mm probe diameter, 20 kHz frequency, 750 W power) at amplitudes of 40%, 50%, and 60% for 30-, 60-, and 90 min. The temperature did not exceed 60 °C using an external water bath for temperature control, and there was an intermittent sonication cycle of 4 s on and 3 s off. After extraction, centrifugation was performed at 8000 rpm for 10 min at 4 °C to separate the supernatant, which was neutralized to pH 7 then mixed with 95% ethanol (1:2, *v*/*v*) and incubated at 4 °C overnight. The crude extract precipitate was collected by centrifugation, neutralized to pH 7, dried at 65 °C in a vacuum oven, and stored at −18 °C until further use.

### 2.6. Experimental Design

The optimization of crude β-glucan extracted from *Lentinula edodes* was conducted using response surface methodology (RSM). A three-factor, three-level Box–Behnken design (BBD) was employed to evaluate the effects of key extraction parameters on β-glucan yield. For the HW and HA extraction methods, the independent variables included solid-to-liquid ratio (1:10–1:30 *w*/*v*), extraction temperature (70–90 °C), and extraction time (30–90 min). For the UW and UA extraction methods, the temperature factor was replaced with ultrasonic amplitude (40–60%) to better reflect the ultrasound conditions. All experimental runs, including center points, were performed in triplicate. The β-glucan content (g/100 g) was used as the response variable (Table 1 and Table 2).

Statistical analyses were conducted with Design Expert 10 software (trial version; Stat-Ease Inc., Minneapolis, MN, USA). The experimental data were fitted to a quadratic model that corresponded with the responses. The standard representation of the quadratic equation is indicated in Equation (1):

*Y* = *b*_0_ + ∑^(k)i=1^
*b*_i_*x*_i_ + ∑^(k)i=1^ ∑^(k)j=i^
*b*_ij_*x*_i_*x*_j_ + ∑^(k)i=1^
*b*_ii_*x*_i_^2^ (1)

where *Y* is the dependent variable; *b*_0_ is the constant; and *b_i_*, *b_ii_*, and *b_ij_* are coefficients estimated by the model. *x_i_* and *x_j_* are the levels of the independent variables.

### 2.7. Measurement of β-Glucan Content

β-glucans were determined using the K-YBGL β-glucan Assay Kit (Yeast and Mushrooms) (Megazyme, Bray, Ireland) [15]. This enzymatic test is a quantitative method to determine 1,3-1,6-β-d-glucan in mushroom and mycelial products, and yeast and fungal preparations. This method involved two separate analyses of the food sample. The initial procedure was the hydrolysis of total glucans by exposing the sample to sulfuric acid to hydrolyze α-glucans (starch and simple polysaccharides) and β-glucans, followed by treatment with exo-1,3-glucanase to reach the complete hydrolysis of the β-glucan. The sample was analyzed to quantify the total glucan concentration of glucose. The secondary analysis involved hydrolyzing samples with Amyloglucosidase and subsequently measuring the glucose content in the digested sample at 510 nm using a spectrophotometer (Thermo Scientific™ GENESYS™ 180, Waltham, MA, USA) to determine the α-glucan concentration. The β-glucan content was determined by the Mega-Calc™ Excel spreadsheet supplied by Megazyme, calculated by deducting α-glucan from the total glucan concentration.

### 2.8. Scanning Electron Microscopy (SEM) Analysis

The four samples were coated with a thin layer of gold under reduced pressure and investigated with a Scanning Electron Microscope (SEM, JSM-IT300, JEOL Ltd., Tokyo, Japan) at a 3 kV acceleration voltage, as well as image magnifications of 1500×.

### 2.9. Swelling Power Determination

According to the method in [16], dry samples of 0.1 g were dissolved in 10 mL of phosphate-buffer saline at different pH values (3, 4, and 6.5) and incubated for 2 h at 37 °C. Then, the sample was centrifuged at 7000 rpm for 15 min and. the residue was collected and weighed. Swelling power is the gain in weight and was calculated by this formula in Equation (2):

Swelling power = (W_2_ − W_1_)/W_1_ × 100 (2)

### 2.10. Fourier-Transform Infrared (FT-IR) Spectroscopy

FTIR spectra were acquired utilizing a JASCO FT/IR 4700 Spectrometer ( JASCO Inc., Easton, MD, USA) operated in KBr mode. All data were collected in the region from 400 to 4000 cm^−1^.

### 2.11. Antioxidant Activity Assays

#### 2.11.1. ABTS Radical Scavenging Activity

The ABTS radical scavenging activity was assessed following the procedure provided by [17]. A stock solution of ABTS+ was produced by combining a 7 mM ABTS solution with a 2.45 mM potassium persulfate solution in a 1:1 ratio and allowed to incubate in the dark at room temperature for 16 h. The ABTS+ stock solution was diluted with distilled water to achieve an absorbance of 0.7 ± 0.02 at 734 nm. After that, 50 µL of sample extract (0.1 g/10 mL) was combined with 100 µL of ABTS solution. Absorbance was recorded after 8 min at 734 nm and determined by using a 96-well microplate reader. The radical scavenging activity was quantified as a percentage in accordance with Equation (3):

%Scavenging activity = (Acontrol − Asample)/Acontrol × 100(3)

#### 2.11.2. DPPH Radical Scavenging Activity

The radical scavenging activity of β-glucan was conducted by using the method in [18]. A 0.2 mM solution of DPPH radical in 70% methanol was produced, and 160 µL of this solution was combined with 40 µL of an aqueous solution containing β-glucan (0.1 g/10 mL). The absorbance of the solutions at 517 nm was determined by using a 96-well microplate reader. Methanol was used as a control, while Trolox was used as a standard. The capability to scavenge the DPPH was calculated using the following Equation (3):

#### 2.11.3. Ferric-Reducing Antioxidant Power (FRAP) Assay

The total antioxidant activity of the polysaccharides was investigated by conducting a FRAP assay by measuring the ferric-reducing power ability of each extract [19]. The working FRAP reagent was produced by combining 10 mL of 300 mM acetate buffer of pH 3.6 with 1 mL of 10 mM TPTZ in 40 mM HCl and 1 mL of 20 mM FeCl_3_·6H_2_O. FRAP reagents were heated to 37 °C. We mixed 150 µL of freshly produced FRAP reagent with 50 μL sample extracts (0.1 g/10 mL). The mixture was incubated at 37 °C for 10 min in a water bath. The absorbance was measured at 593 nm against a reagent blank after 4 min. The FRAP value was calculated as micromoles (or millimoles) of Fe^2+^ equivalent per 100 g of sample using the Fe^2+^ calibration curve.

### 2.12. Total Phenolic Compound (TPC)

Total phenolic content (TPC) in each extract was determined according to the method of [20]. A total of 25 µL of each extract (0.1 g dissolved in 10 mL) was mixed with 125 µL of 0.2 M Folin–Ciocalteu reagent after standing for 10 min in the dark, and 125 µL of 7.5% Na_2_CO_3_ was added to the mixture after standing for 30 min in the dark. The mixture was centrifuged at 13,400× *g* for 5 min. The absorbance was measured at 750 nm and determined by using a 96-well microplate reader and TPC was expressed as gallic acid equivalents (GAEs).

### 2.13. α-Amylase Inhibition Activity

The alpha-amylase inhibitory activity was evaluated based on a published method with slight modifications [21]. The mushroom extracts of 50 μL (containing 0.0625 to 2.0 mg/mL) were added to 50 μL of α-amylase solution (1 U/mL, 20 mM sodium phosphate buffer with 6.7 mM NaCl (pH 6.9)) and incubated at 37 °C for 15 min. Then 100 μL of 1% of starch solution was added to the mixture and incubated at 37 °C for 30 min. After that, we added 100 μL of 96 mM 3,5-dinitrosalicylic acid (DNS) and incubated at 100 °C for 3 min. The reaction solution absorbance was determined at 540 nm using a microplate reader. Acarbose was used as a positive control. The α-glucosidase inhibitory activity was calculated as follows:

%Inhibition = (Acontrol − Asample)/Acontrol × 100(4)

### 2.14. α-Glucosidase Inhibition Activity

The α-glucosidase inhibitory effect of the samples was measured with slight modification [22]. A total of 50 μL of the samples (containing 0.0625 to 2.0 mg/mL) was added to 50 μL of α-glucosidase solution (1 U/mL, dissolved in 0.1 M pH 6.8 phosphate buffer) and incubated at 37 °C for 15 min. Then 50 μL of pNPG solution (4 mM, dissolved in 0.1 M pH 6.8 phosphate buffer) was added to the mixture and incubated at 37 °C for 30 min. The reaction solution absorbance was determined at 405 nm using a microplate reader. Acarbose was used as a positive control. The α-glucosidase inhibitory activity was calculated as follows in Equation (4):

### 2.15. Statistical Analysis

The statistical analyses of the results were conducted by SPSS software (version 23.0, SPSS Inc., Chicago, IL, USA) and were presented as mean values with standard deviations. Duncan multiple comparison tests were used (*p* < 0.05) to evaluate the differences between the means.

## 3. Results and Discussion

### 3.1. Extraction Optimization by RSM

#### 3.1.1. Extraction Efficiency and Yield of β-Glucan

This study analyzed four different extraction methods of β-glucan from *L. edodes*, including HW, HA, UW, and UA. The initial β-glucan content in the dried shiitake mushroom powder was 11.87 ± 1.31 g/100 g dry weight. Among all methods, the UA extraction demonstrated the highest extraction yield (45.17 ± 0.35%), followed by UW (33.15 ± 3.51%). In contrast, HA and HW showed considerably lower yields of 25.10 ± 5.40% and 14.52 ± 0.82%, respectively, as presented in Table 3 and Table 4.

The β-glucan concentration in the crude extracts varied significantly among the extraction methods. UW yielded the highest β-glucan content at 34.47 ± 2.96 g/100 g dry extract, followed by HA (33.49 ± 2.91 g/100 g dry extract), UA (32.77 ± 3.81 g/100 g dry extract), and HW (30.72 ± 0.70 g/100 g dry extract). Notably, the UW method not only provided the highest β-glucan concentration but also exhibited the lowest coefficient of variation (CV = 3.54%), indicating the high consistency and reliability of the process. To comprehensively evaluate extraction efficiency, absolute β-glucan recovery was calculated as the product of extraction yield and β-glucan content, relative to the initial β-glucan content in the mushroom (11.87 g/100 g dry weight). UA achieved the highest recovery, extracting approximately 61.82% of the initial β-glucan, followed by UW (45.26%), HA (31.47%), and HW (19.62%). Additionally, the β-glucan content-to-extraction yield ratio was used as an index of extraction. UW showed the highest ratio (2.44), suggesting a more concentrated and purer β-glucan extract. This was followed by HW (2.26), HA (2.21), and UA (2.18), respectively. These findings underscore the potential of UW extraction for producing high β-glucan extracts with consistent efficiency.

#### 3.1.2. Predicted Model and Statistical Analysis

The response surface methodology (RSM) was applied to study the factors affecting extraction yield (EY) and β-glucan content (BGC) to determine the optimal extraction conditions. According to the analysis, the models for all four methods were appropriate; the response variable and the test variables were associated with the following second-order polynomial Equations (5)–(12).

HW: EY = 9.74 + 2.15A + 2.31B + 0.06C + 1.64AB + 1.09AC − 0.54BC − 1.80A2 + 0.27B2 − 0.70C2(5)

HA: EY = 10.08 + 3.00A + 2.38B − 0.80C + 0.09AB + 0.16AC − 1.73BC + 0.98A2 + 6.45B2 + 1.82C2(6)

UW: EY = 20.09 + 9.62A + 5.06B + 0.77C + 3.07AB + 1.28AC + 2.90BC − 4.26A2 + 1.67B2 − 0.67C2(7)

UA: EY = 29.32 + 11.54A + 5.89B − 2.50C − 1.08AB + 4.34AC + 0.45BC − 1.32A2 + 1.37B2 − 2.15C2(8)

HW: Y = 27.43 + 1.74A + 2.46B − 0.49C + 0.87AB − 0.06AC − 1.20BC − 0.71A^2^ − 2.16B^2^ − 0.29C^2^ (9)

HA: Y = 32.11 + 13.39A + 1.19B − 1.22C + 1.19AB + 1.45AC − 1.70BC − 3.54A^2^ − 4.31B^2^ − 3.86C^2^ (10)

UW: Y = 30.03 + 2.82A + 1.00B − 1.55C + 2.26AB − 1.51AC − 0.21BC − 2.07A^2^ − 0.47B^2^ − 2.77C^2^ (11)

UA: Y = 29.94 + 4.63A − 1.52B − 3.75C − 0.70AB − 0.91AC − 0.18BC − 3.01A^2^ − 3.88B^2^ − 3.64C^2^ (12)

The coefficient of determination (R^2^) ranged from 0.9188 to 0.9728, and the adjusted R^2^ values were all above 0.81, indicating that the models could explain the variability in the data well. A coefficient of variation (C.V.) lower than 15% in all models reflected the precision of the experiments, and a non-significant lack of fit (*p* > 0.05) confirmed the statistical adequacy of the models [23]. The corresponding data are shown in Table 5 and Table 6.

Among the models, the UW method showed the highest accuracy in β-glucan content (R^2^ = 0.9622, Adj R^2^ = 0.9136, C.V. = 3.54%), followed by the UA method, which confirms the effectiveness of ultrasound in enhancing extraction efficiency. Variable A (ratio) significantly influenced all extraction methods, demonstrating the importance of concentration in β-glucan extraction. In the HW and HA methods, variable B (temperature) also significantly affected the yield, while variable C (time) showed no clear effect. For the UW and UA methods, the models were more complex, with there being significance of the quadratic terms (A^2^, C^2^) and interaction terms (AB, AC), especially in the UW method. This indicates the impact of cavitation forces from ultrasound, which affect cell structure and increase diffusion. This structural change, caused by ultrasonic cavitation, enhances the release of compounds from plant cells by creating micro-fractures on the surface of the samples [9]. Based on actual experimental data, the UW method yielded the highest amount of β-glucan, followed by UA, HA, and HW, respectively. Although the UA model provided the highest sum of squares, reflecting the overall influence of the factors in the model, when considering the actual yields, the UW method clearly produced the highest β-glucan content.

#### 3.1.3. Optimization and Comparative Analysis of Extraction Parameters Affecting β-Glucan Content

**Figure 1:**
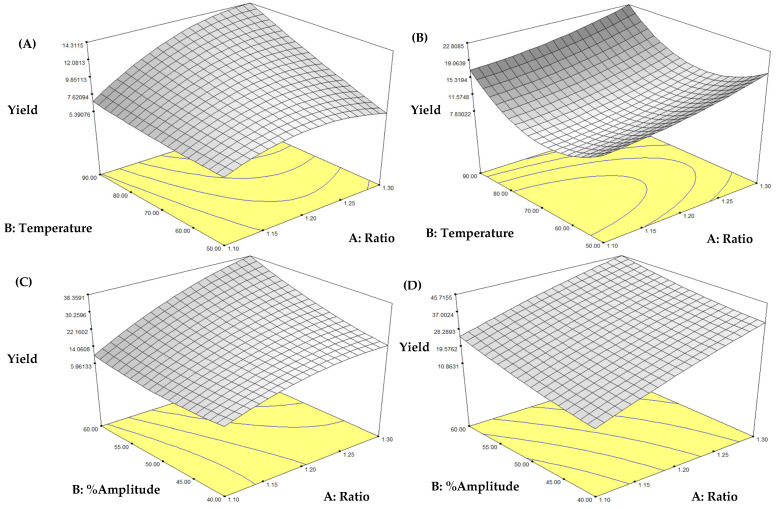
Response surface plots showing the effects of extraction parameters on yield extraction from *L. edodes* under different extraction methods: (**A**) hot water, (**B**) hot alkaline, (**C**) ultrasound-assisted water, and (**D**) ultrasound-assisted alkaline extraction.

**Figure 2:**
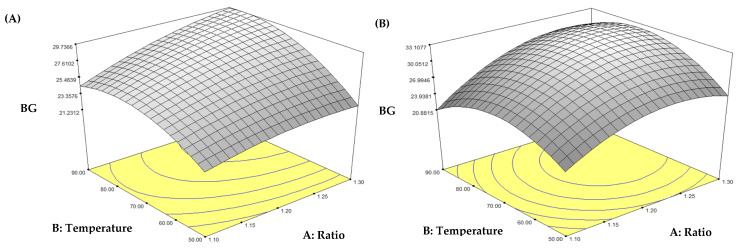

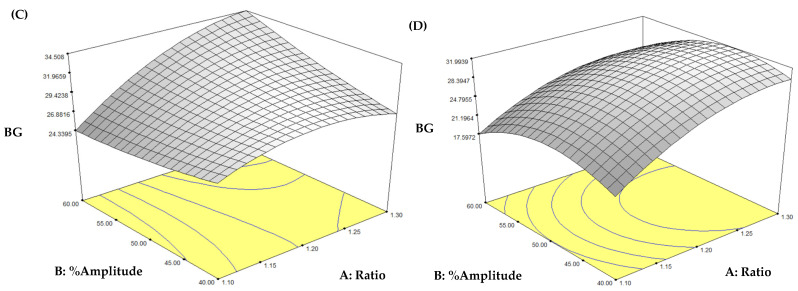
Response surface plots showing the effects of extraction parameters on β-glucan content from mushroom under different extraction methods: (**A**) hot water, (**B**) hot alkaline, (**C**) ultrasound-assisted water, and (**D**) ultrasound-assisted alkaline extraction.

The results revealed that the extraction technique significantly affected extraction efficiency. Among the methods investigated, UW exhibited the highest yield (35.16%), outperforming conventional thermal methods. UA also showed high efficiency, with a yield of 32.97%. In contrast, conventional hot water (HW) and hot alkaline (HA) extractions resulted in lower yields of 12.94% and 12.59%, respectively (Figure 1). Analysis of β-glucan content further confirmed that the extraction method had a significant impact on both the yield and quality of the polysaccharides obtained. UW yielded the highest β-glucan content at 34.51 ± 0.82 g/100 g dry extract, followed by HA (33.10 ± 0.79 g/100 g), UA (31.99 ± 0.96 g/100 g), and HW (29.74 ± 0.53 g/100 g) (Figure 2). These findings highlight the effectiveness of ultrasound-assisted methods in enhancing both the efficiency and quality of β-glucan extraction from mushrooms.

These results align with previous studies indicating that ultrasound can significantly enhance the extraction of polysaccharides from mushrooms. This phenomenon is attributed to the cavitation mechanism, in which the formation and violent collapse of microbubbles in the liquid medium generate localized shear forces and mechanical shock, leading to the disruption of cell walls and facilitating the release of β-glucan [24]. The effect of alkaline conditions on β-glucan extraction was evident when considering the high yield obtained from HA, second only to the UW method. This result can be explained by the ability of alkaline solutions to disrupt hydrogen bonds and ester linkages between β-glucan, proteins, and chitin in the mushroom cell wall [25]. However, the UA method yielded lower β-glucan than both UW and HA, which may have resulted from the partial degradation of β-glucan due to the combined effects of ultrasound and alkaline conditions. This phenomenon is consistent with reports of polysaccharide degradation under ultrasonic treatment in the presence of high alkali concentrations [26]. Additionally, previous studies suggest that a small amount of NaOH is sufficient to effectively disrupt the cell wall, while excessive NaOH may negatively impact extraction yield. A NaOH concentration of approximately 0.52% was found to be optimal [27]. Temperature and ultrasound intensity are key factors influencing β-glucan extraction. Conventional extraction methods (HW and HA) rely primarily on temperature, with the optimal range found to be approximately 80–90 °C for both techniques. In contrast, ultrasound-assisted extraction methods (UW and UA) rely on amplitude intensity as a key variable, with optimal amplitudes at 50–60% for UW and 50–55% for UA. Experimental data showed that excessively high ultrasound amplitudes (>60%) in the UA method resulted in reduced β-glucan yield, likely due to degradation of the β-glucan molecule under overly harsh conditions, particularly when combined with alkaline treatment. Although alkaline conditions alone can enhance β-glucan release compared to water, excessive ultrasound intensity may lead to polysaccharide breakdown [28]. Furthermore, the optimal solvent-to-sample ratios varied slightly between methods. HW and HA required relatively higher ratios (approximately 1:25 to 1:30), whereas UW and UA required slightly lower ratios (approximately 1:20 to 1:25). This can be explained by the mechanical action of ultrasound-induced cavitation, which generates microbubbles that collapse violently, disrupting cell walls and enhancing solvent penetration into the cell–matrix. This process improves extraction efficiency by facilitating the diffusion of the solvent into cellular structures, thereby requiring less solvent to achieve comparable yields of β-glucan [29].

#### 3.1.4. Validation of Model

The efficiency of the response surface model equations in predicting the optimum response was determined by validation experiments conducted under significantly modified optimal conditions. The experimental yields and predicted maximum yields of β-glucan extracted from *L. edodes* were compared, as shown in Table 7. The strong correlation between these numbers establishes the validity of the RSM models. The strong correlation between predicted and experimental outcomes suggests that the response models were adequate for characterizing the optimization process. The results indicate that the fitted models (Equations (5)–(12)) were accurate and reliable in predicting β-glucan yields under the investigated conditions.

### 3.2. SEM Analysis

**Figure 3:**
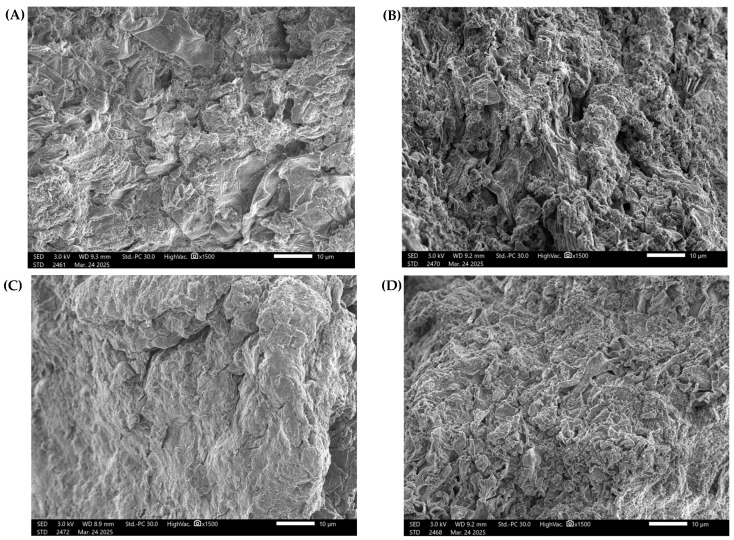
Scanning Electron Microscopy (SEM) images of crude polysaccharides extracted using different extraction methods: (**A**) HW, (**B**) HA, (**C**) UW, and (**D**) UA. All images were taken at 1500× magnification, scale bar = 10 µm.

Scanning Electron Microscopy (SEM) showed significant differences in the morphology of crude polysaccharides extracted by different techniques. Samples analyzed by SEM were extracted under optimized conditions. These conditions corresponded to the RSM-optimized protocol for each extraction method (HW, HA, UW, UA), as shown in Table 7. These variations show the impact of the extraction methods on the microstructural characteristics of the resultant β-glucan, as shown in Figure 3.

The crude polysaccharides extracted via HW exhibited a relatively smooth surface with thin layered structures and irregularly distributed cracks and small voids. This observation aligns with previous studies indicating that hot water extraction tends to preserve the fundamental polysaccharide structure, although it may not efficiently disrupt the cell–matrix to fully release crude polysaccharides [11]. In contrast, crude polysaccharides extracted under HA displayed a rough and porous surface with numerous large cavities spread throughout the structure. This is likely due to the alkaline solution’s capacity to disrupt hydrogen bonding, leading to swelling and significant structural changes in the polysaccharide matrix [30]. While this method yielded a high amount of crude polysaccharide, it may also have induced substantial molecular alterations. UW resulted in a more homogeneous morphology with a higher density and a smoother surface, showing only minor surface fractures. This can be attributed to the cavitation-induced shear forces generated by ultrasound, which efficiently disrupted cell walls and facilitated the release of intracellular components while preserving chemical integrity [31]. These characteristics correspond with the highest crude polysaccharide yield observed among the methods tested. Meanwhile, β-glucan obtained via UA presented morphological features that combined aspects of both HA and UW methods. The surface was notably rough and porous, like HA, but with a more compact and ordered structure. Clear aggregation of particles was observed, possibly resulting from the combined effects of alkali-induced swelling and ultrasound-enhanced cellular disruption [32]. These morphological differences are critically important, as they may directly affect the physicochemical properties and bioactivities of the extracted crude polysaccharides.

### 3.3. FTIR Analysis

**Figure 4:**
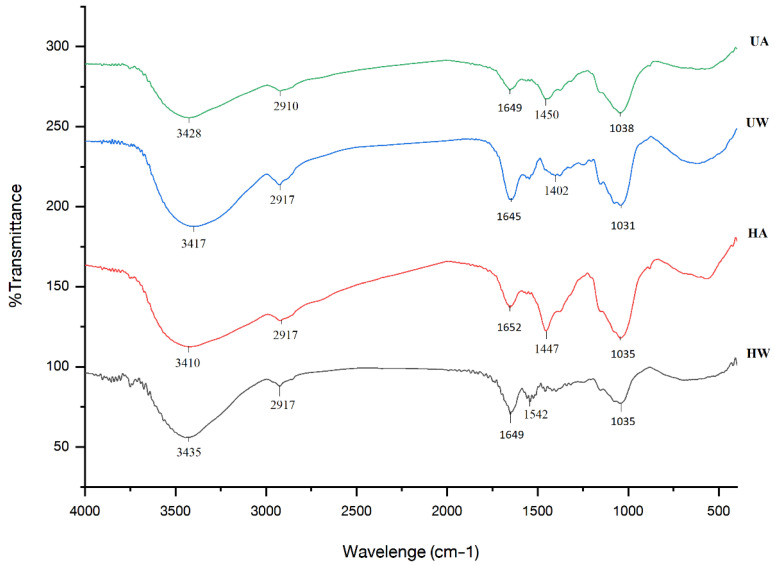
FTIR spectrum of isolated crude polysaccharides from different conditions.

The FTIR spectra revealed characteristic absorption bands indicative of the structural features of crude polysaccharides extracted under different conditions. All four extraction methods were extracted under optimized conditions. As shown in Table 7, HW, HA, UW, and UA exhibited generally similar spectral patterns. However, observable differences in peak intensities and slight shifts in band positions were noted, as shown in Figure 4. These variations suggest subtle structural modifications influenced by the extraction conditions.

A broad band observed around 3410–3435 cm^−1^ corresponds to O–H stretching vibrations in hydroxyl groups, indicative of intra- and intermolecular hydrogen bonding typically present in polysaccharide structures. Variations in intensity and slight shifts in this region reflect differences in hydrogen bonding patterns among crude polysaccharides extracted using different methods. Particularly, the UW method showed a distinct peak at 3417 cm^−1^, suggesting a higher hydroxyl group content. The band observed at approximately 2910–2917 cm^−1^ is attributed to the C–H stretching of CH_2_ groups in the glucopyranose rings. All extraction methods preserved this structural feature, confirming the retention of the carbohydrate backbone. The peak at 1642–1652 cm^−1^ corresponds to bound water, while the band at 1447–1450 cm^−1^ is related to CH_2_ bending vibrations. A key indicator of crude β-glucan is the peak observed at 1031–1038 cm^−1^, which is associated with β-(1→3) glycosidic linkages [33]. Among the methods, the UW-extracted crude polysaccharides exhibited the highest intensity at 1031 cm^−1^, suggesting a higher yield and better preservation of the crude β-glucan structure [34], especially the β-(1→3) backbone—an essential structural motif known for its potent biological activity in mushrooms. Mushroom-derived β-glucans differ from cereal-derived ones by featuring a β-(1→3)-linked backbone with β-(1→6) branches, which are crucial for their immunomodulatory and anticancer properties [35]. The higher intensity at 1031 cm^−1^ in the UW method implies the effective preservation of this biologically active structure.

### 3.4. Swelling Capacity of Polysaccharides

**Figure 5:**
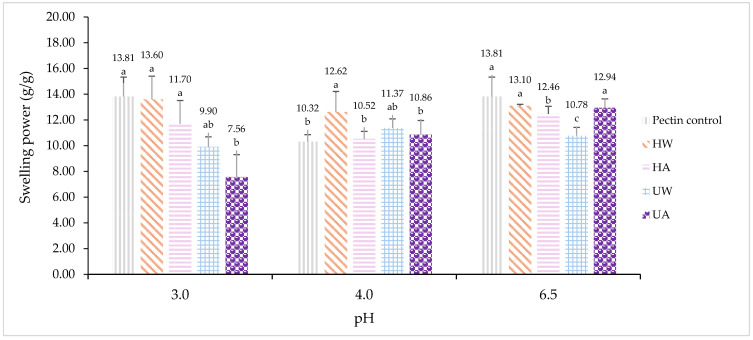
Swelling power of crude polysaccharides extracted from *L. edodes* using different extraction methods. Different letters (a–c) above the bars indicate significant differences (*p* < 0.05) according to Duncan’s multiple range test.

The swelling capacity of crude polysaccharides extracted from *L. edodes* using various extraction methods (conditions summarized in Table 7) was evaluated at pH 3.0, 4.0, and 6.5, representing different segments of the human gastrointestinal tract. The results are presented in Figure 5.

Swelling capacity, expressed in g/g, was compared against pectin. The pH values represent gastric conditions (pH 3.0), the gastric–intestinal transition (pH 4.0), and the small intestinal environment (pH 6.5) [36]. Swelling behavior is a critical functional property of β-glucan, as it directly influences gel formation and viscosity in the digestive tract, which are closely associated with the delayed absorption of glucose and postprandial glycemic control [37]. Our findings revealed that the extraction method significantly influenced the swelling behavior of crude polysaccharides in simulated gastrointestinal conditions. HW yielded crude polysaccharides with the most stable and consistently high swelling capacities across all tested pH levels. This observation aligns with reports indicating that hot water extraction helps preserve the structural integrity of crude polysaccharide molecules, which are crucial for gelation and swelling properties [38]. Especially, crude polysaccharides extracted by HW performed comparably or even superiorly to standard pectin under certain pH conditions, which is significant given that pectin is a widely recognized polysaccharide for glycemic modulation [39]. The sustained swelling capacity of HW-extracted crude polysaccharides across different pH levels suggested the potential for maintaining physiological effectiveness throughout the digestive process, a major advantage over pectin, which exhibited a significant decrease in swelling at pH 4.0. The preservation of swelling and gel-forming abilities throughout the gastrointestinal tract is crucial for delaying carbohydrate digestion and glucose absorption. HA also resulted in favorable swelling behavior, second only to HW, demonstrating a similar pattern with slightly reduced values. The molecular weight of crude polysaccharides was found to vary depending on the extraction method. Generally, the molecular weight of crude polysaccharides from *L edodes* ranges from 1.16 × 10^3^ to 6.842 × 10^6^ Da [40]. In this study, the sample extracted using hot water exhibited a molecular weight of approximately 7.8 × 10^5^ Da [41], whereas the alkaline-extracted sample showed a lower molecular weight of 9.6 × 10^4^ Da [42]. This finding is consistent with previous reports indicating that alkaline extraction can induce the partial hydrolysis of crude polysaccharide structures, thereby reducing their molecular weight. A decrease in molecular weight may consequently result in the lower water-holding capacity of the polysaccharides [43]. Nonetheless, HA maintained a relatively strong performance, particularly at pH 6.5, which is critical for glucose absorption in the small intestine. Interestingly, crude polysaccharides extracted via ultrasound-assisted methods (UW and UA) exhibited distinct swelling profiles. UA demonstrated the lowest swelling at pH 3.0, followed by a pronounced increase at pH 4.0 and pH 6.5. This shift could be attributed to ultrasound-induced structural modifications, especially when combined with alkaline conditions. Such changes are in line with previous studies showing that ultrasound processing can alter polysaccharide structures, leading to lower-molecular-weight fractions and changes in the degree of polymerization, thereby affecting swelling behavior [44]. The differences in swelling capacity observed among extraction methods are clinically relevant for glycemic control. High swelling at gastric pH (3.0) has been associated with enhanced satiety, delayed gastric emptying, and reduced nutrient absorption. Meanwhile, sustained swelling at pH 6.5 is critical for slowing down enzymatic digestion and glucose uptake in the small intestine [45]. Therefore, crude polysaccharides extracted from HW demonstrate the highest potential for comprehensive postprandial glycemic management throughout digestion. Additionally, the low standard deviation observed at pH 6.5 (± 0.11 g/g) for HW indicates consistent swelling behavior in intestinal conditions, which is an advantage for developing functional foods or supplements aimed at blood glucose regulation. This consistency facilitates accurate dosing and reliable performance in clinical applications.

### 3.5. Antioxidant Activity

The antioxidant activity and total phenolic content (TPC) of crude polysaccharides isolated from *L. edodes* with four different extraction techniques (conditions summarized in Table 7) have been evaluated, as shown in Table 8.

The antioxidant activity of the extracted samples was evaluated using three complementary assays (ABTS, DPPH, and FRAP), and the results showed significant variation among extraction methods. As shown in Table 7, the hot alkaline (HA) extract exhibited the highest ABTS radical scavenging activity (5.99 ± 0.15 μmol TE/g), followed by hot water (HW) at 4.52 ± 0.08 μmol TE/g. Ultrasound-assisted extracts (UW and UA) demonstrated lower activities at 3.82 ± 0.02 and 3.12 ± 0.17 μmol TE/g, respectively. A similar trend was observed for DPPH activity, where HA yielded the highest activity (3.73 ± 0.02 μmol TE/g), followed by UA (3.36 ± 0.59 μmol TE/g), HW (3.27 ± 0.19 μmol TE/g), and UW (3.12 ± 0.35 μmol TE/g). FRAP results were also the highest in the HA group (0.42 ± 0.02 μmol TE/g), further supporting the effectiveness of alkaline-assisted extraction in enhancing antioxidant potential [46]. To further explore antioxidant mechanisms, total phenolic content (TPC) was assessed to evaluate the contribution of phenolic compounds. TPC values did not differ significantly among HA (0.27 ± 0.42 mg GAE/g), UA (0.26 ± 0.02 mg GAE/g), and UW (0.25 ± 0.17 mg GAE/g), although HW displayed significantly lower levels (0.21 ± 0.12 mg GAE/g). While the observed TPC values were relatively low, they provided insight into the potential release of bound phenolics, particularly under alkaline conditions, which may have facilitated the cleavage of ester and hydrogen bonds between phenolics and cell wall polysaccharides. [47]. It is important to acknowledge that the TPC assay was performed on crude extracts, not on isolated polysaccharidic fractions. Therefore, the detected phenolics likely include both free and polysaccharide-bound forms. Previous studies have shown that polysaccharides are able to bind to phenolic acids and to tyrosine by esterification [48]. However, loosely associated phenolics would be lost during the ethanol precipitation and washing steps. Interestingly, despite UW producing the highest β-glucan content, its antioxidant capacity was among the lowest, suggesting that the antioxidant potential of the extracts was not solely dependent on β-glucan quantity, but also on structural and compositional factors. Collectively, the results suggest that phenolics, though present in small amounts, may contribute to the observed antioxidant activity, particularly in HA and UA samples. However, due to the complexity of extract composition, further structural analysis is required to elucidate the specific antioxidant mechanisms.

### 3.6. Inhibitory Activities of α-Amylase and α-Glucosidase

**Figure 6:**
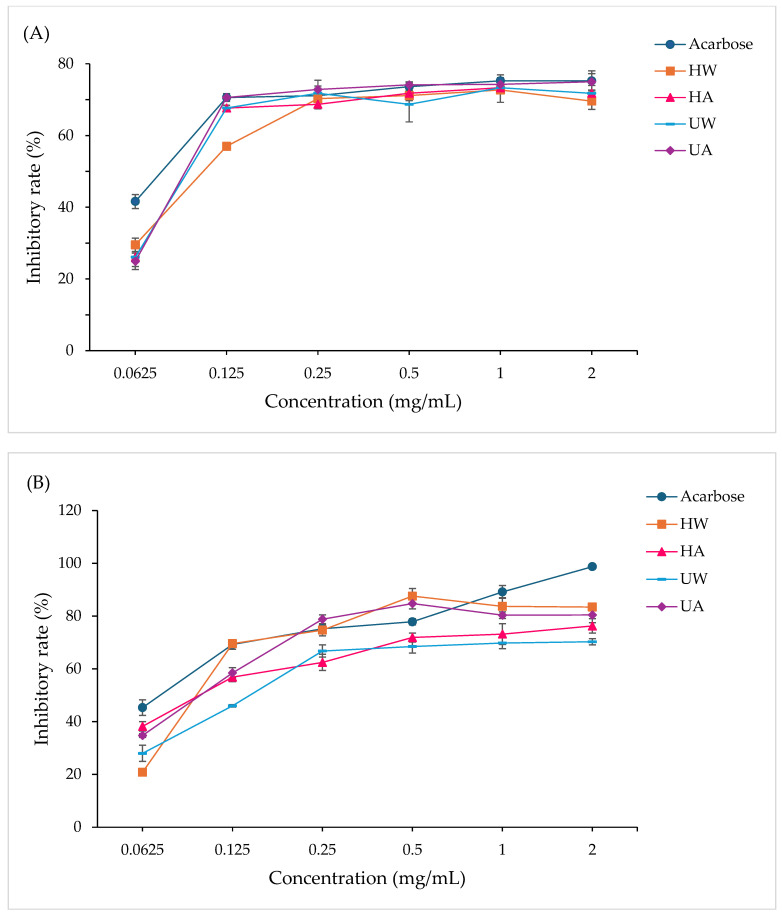
Enzyme inhibitory activities of crude polysaccharides. (**A**) α-Amylase inhibition. (**B**) α-Glucosidase inhibition.

Crude polysaccharides extracted from *L. edodes* using various extraction methods exhibited significantly different inhibitory activities against α-amylase and α-glucosidase. All four extraction methods were extracted under optimized conditions. As shown in Table 7, these enzymatic inhibitions are of relevance to the potential for delaying carbohydrate digestion and regulating postprandial blood glucose levels. The results demonstrate method-specific inhibition patterns, as shown in Figure 6.

The inhibitory activity of the crude polysaccharide fractions against α-amylase and α-glucosidase was evaluated to explore their potential antidiabetic properties. Notably, these fractions were not composed solely of β-glucans but also contained other co-extracted components, such as polyphenols and low-molecular-weight substances, which may have contributed to or interfered with enzyme inhibition. For α-amylase, the UA extract exhibited the strongest inhibition (IC_50_ = 1.46 mg/mL), followed by UW (IC_50_ = 1.59 mg/mL) and HA (IC_50_ = 1.64 mg/mL). The HW extract showed the weakest inhibition (IC_50_ = 1.85 mg/mL). Although all extracts displayed lower potency than the reference drug acarbose (IC_50_ = 0.11 mg/mL), both UA and UW demonstrated significantly better inhibitory effects compared to the conventional extraction methods. This may be partially attributed to the structural changes induced by ultrasound, such as molecular size reduction and increased surface area, which enhance interaction with enzymes. However, the influence of non-polysaccharide constituents in the crude extracts should also be considered when interpreting the observed inhibitory activities, as various co-extracted compounds may contribute to the measured bioactivities. The dose response evaluation showed that at a low concentration (0.0625 mg/mL), inhibition was minimal for all extracts. At 0.125 mg/mL, the UA extract exhibited marked improvement, approaching the activity of acarbose. At concentrations above 0.5 mg/mL, inhibition plateaued 70–75%, suggesting saturation of the enzyme binding sites. These findings suggest that both extraction method and extract composition influence inhibitory efficiency. In contrast, α-glucosidase inhibition presented a different pattern. UA again showed the highest activity (IC_50_ = 1.21 mg/mL), followed by HA (1.65 mg/mL) and HW (1.60 mg/mL), while UW exhibited the lowest inhibition (IC_50_ = 2.49 mg/mL), despite having the highest β-glucan content in previous analyses. This suggests that enzyme inhibition is not directly correlated with β-glucan concentration alone. Interestingly, at low concentrations (0.0625 mg/mL), HA showed the most pronounced inhibition, potentially due to residual phenolic compounds or other bioactives co-extracted under alkaline conditions. As concentration increased, UA and HW exhibited stronger inhibition, with HW reaching 87.6% inhibition at 0.5 mg/mL before slightly declining at higher doses. Acarbose showed a consistent dose-dependent inhibition, surpassing 99% at 2 mg/mL. These results underscore the complex relationship between extraction conditions, extract composition, and bioactivity. Although β-glucans are well-documented inhibitors of carbohydrate-hydrolyzing enzymes, the presence of polyphenols and other minor constituents in the crude extracts complicates the direct attribution of activity to β-glucans alone. The disparity in inhibition patterns—particularly the weak α-glucosidase inhibition by the UW extract despite its high β-glucan content—supports previous findings that structural properties such as branching degree, molecular weight, and glycosidic linkages affect enzymatic interactions [49]. Additionally, ultrasound under alkaline conditions may introduce functional group modifications that enhance enzyme binding [50]. Therefore, while β-glucans likely contribute to the inhibitory effects, the potential synergistic or antagonistic roles of co-extracted phenolics and other constituents should not be overlooked. Future studies using purified β-glucans and phenolic-free fractions would be beneficial to delineate the specific contributions of individual components.

## 4. Conclusions

The present study revealed that the extraction method significantly influenced not only the yield but also the physicochemical properties and bioactivities of crude polysaccharides from *L. edodes*. The UW method, while giving the highest β-glucan content, did not exhibit the strongest bioactivity, suggesting that extraction efficiency alone does not dictate functionality. HW-extracted samples showed the most consistent swelling power across a pH range, indicating superior structural preservation. Despite lower β-glucan content, the HA and UA extracts displayed enhanced antioxidant and enzyme inhibitory activities, respectively, highlighting the role of co-extracted compounds such as phenolics and the impact of extraction conditions on bioactivity. Notably, the UA extract inhibited α-glucosidase more effectively than UW, underscoring that molecular structure, branching, and possible chemical modifications under alkaline conditions influence bioactivity more than β-glucan content alone. Furthermore, the addition of % recovery, statistical validation, and error analysis strengthened the reliability of the optimization model. These findings highlight the importance of selecting extraction methods based on targeted bioactivities rather than yield alone. The study also acknowledges the limitations of crude extracts and proposes future work on purifying polysaccharide fractions and performing detailed structural analyses, including methylation and NMR, to confirm their specific bioactive roles.

## Figures and Tables

**Table 1:** Hot water and hot alkaline extraction method factors used in the BBD matrix for β-glucan content.

Run	A: Solid–Liquid Ratio (g/mL)	B: Extraction Temperature (°C)	C: Extraction Time (h)
1	1:10 (−1)	50 (−1)	60 (0)
2	1:30 (1)	50 (−1)	60 (0)
3	1:10 (−1)	90 (1)	60 (0)
4	1:30 (1)	90 (1)	60 (0)
5	1:10 (−1)	70 (0)	30 (−1)
6	1:30 (1)	70 (0)	30 (−1)
7	1:10 (−1)	70 (0)	90 (1)
8	1:30 (1)	70 (0)	90 (1)
9	1:20 (0)	90 (1)	30 (−1)
10	1:20 (0)	90 (1)	30 (−1)
11	1:20 (0)	50 (−1)	90 (1)
12	1:20 (0)	90 (1)	90 (1)
13	1:20 (0)	70 (0)	60 (0)
14	1:20 (0)	70 (0)	60 (0)
15	1:20 (0)	70 (0)	60 (0)
16	1:20 (0)	70 (0)	60 (0)
17	1:20 (0)	70 (0)	60 (0)

**Table 2:** Ultrasound-assisted water and ultrasound-assisted alkaline extraction method factors used in the BBD matrix for β-glucan content.

Run	A: Solid–Liquid Ratio (g/mL)	B: Amplitude (%)	C: Extraction Time (h)
1	1:10 (−1)	40 (−1)	60 (0)
2	1:30 (1)	40 (−1)	60 (0)
3	1:10 (−1)	60 (1)	60 (0)
4	1:30 (1)	60 (1)	60 (0)
5	1:10 (−1)	50 (0)	30 (−1)
6	1.30 (1)	50 (0)	30 (−1)
7	1:10 (−1)	50 (0)	90 (1)
8	1:30 (1)	50 (0)	90 (1)
9	1:20 (0)	40 (−1)	30 (−1)
10	1:20 (0)	60 (1)	30 (−1)
11	1:20 (0)	40 (−1)	90 (1)
12	1:20 (0)	60 (1)	90 (1)
13	1:20 (0)	50 (0)	60 (0)
14	1:20 (0)	50 (0)	60 (0)
15	1:20 (0)	50 (0)	60 (0)
16	1:20 (0)	50 (0)	60 (0)
17	1:20 (0)	50 (0)	60 (0)

**Table 3:** β-glucan content, extraction yield, and recovery percentage from *L. edodes* by hot water extraction (HW) and hot alkaline extraction (HA).

Run	Hot Water Extraction (HW)			Hot Alkaline Extraction (HA)		
	%Yield-HW	BG-HW (g/100 Dry Extract)	%Recovery	%Yield-HA	BG-HA (g/100 g Dry Extract)	%Recovery
1	5.18 ± 0.99	21.42 ± 1.17	9.34 ± 0.51	13.50 ± 1.97	19.48 ± 6.36	22.16 ± 7.23
2	6.79 ± 0.61	23.75 ± 1.87	13.58 ± 1.07	20.07 ± 3.31	24.45 ± 4.65	41.32 ± 7.86
3	6.34 ± 1.62	23.64 ± 1.79	12.63 ± 0.96	13.19 ± 2.86	21.72 ± 1.20	24.13 ± 1.34
4	14.52 ± 0.82	29.44 ± 0.26	36.01 ± 0.31	21.34 ± 3.58	31.43 ± 5.96	56.49 ± 10.71
5	6.25 ± 0.38	25.46 ± 2.55	13.40 ± 1.34	10.85 ± 3.80	25.40 ± 5.81	23.21 ± 5.31
6	7.75 ± 0.16	28.47 ± 0.36	18.58 ± 0.23	16.17 ± 1.16	28.70 ± 6.01	39.08 ± 8.18
7	4.56 ± 0.89	25.68 ± 0.40	9.86 ± 0.15	9.28 ± 2.28	17.82 ± 0.30	13.94 ± 0.23
8	10.43 ± 1.41	28.46 ± 4.65	25.00 ± 4.08	15.25 ± 0.13	26.94 ± 0.13	34.59 ± 0.16
9	6.47 ± 0.74	23.15 ± 5.18	12.61 ± 2.82	13.56 ± 2.62	22.28 ± 0.70	25.44 ± 0.80
10	12.39 ± 0.77	30.72 ± 0.70	32.06 ± 0.73	25.10 ± 5.40	25.82 ± 3.17	54.59 ± 6.70
11	7.13 ± 0.36	22.36 ± 1.71	13.43 ± 1.03	15.07 ± 4.15	25.47 ± 5.79	32.32 ± 7.35
12	11.04 ± 0.96	26.28 ± 2.12	24.44 ± 1.98	19.66 ± 4.50	22.20 ± 3.74	36.77 ± 6.19
13	9.26 ± 1.23	27.94 ± 4.88	21.79 ± 3.80	9.37 ± 4.70	31.39 ± 1.01	24.76 ± 0.79
14	10.46 ± 2.06	28.01 ± 1.85	24.67 ± 1.63	9.22 ± 1.12	33.49 ± 2.91	26.00 ± 2.26
15	9.26 ± 1.19	26.15 ± 6.22	20.39 ± 4.85	10.46 ± 1.05	33.13 ± 2.30	29.18 ± 2.03
16	10.60 ± 3.00	27.46 ± 3.11	24.50 ± 2.77	10.13 ± 1.84	31.33 ± 2.69	26.71 ± 2.30
17	9.15 ± 2.49	27.60 ± 3.32	21.26 ± 2.56	9.25 ± 1.61	31.25 ± 1.69	24.33 ± 1.31

**Table 4:** β-glucan content, extraction yield, and recovery percentage from *L. edodes* by ultrasound-assisted water (UW) and ultrasound-assisted alkaline (UA).

Run	Ultrasound-Assisted Water (UW)			Ultrasound-Assisted Alkaline (UA)		
	%Yield-UW	BG-UW (g/100 g Dry Extract)	%Recovery	%Yield-UA	BG-UA (g/100 g Dry Extract)	%Recovery
1	7.69 ± 1.18	26.90 ± 0.50	17.43 ± 0.32	11.41 ± 0.50	19.40 ± 1.42	18.65 ± 1.37
2	20.44 ± 0.46	27.12 ± 0.51	46.69 ± 0.88	35.78 ± 0.31	29.73 ± 1.50	89.57 ± 4.52
3	8.44 ± 0.25	25.22 ± 8.55	17.92 ± 6.07	25.12 ± 5.12	17.78 ± 3.07	37.62 ± 6.50
4	33.15 ± 3.51	34.47 ± 2.96	96.23 ± 8.26	45.17 ± 0.35	25.30 ± 0.96	96.22 ± 3.64
5	5.19 ± 0.05	21.52 ± 7.98	9.40 ± 3.48	20.53 ± 0.93	20.70 ± 3.66	35.80 ± 6.33
6	22.24 ± 0.98	31.10 ± 1.52	58.26 ± 2.85	35.80 ± 9.65	32.13 ± 3.17	96.88 ± 9.57
7	5.53 ± 0.32	22.28 ± 1.21	10.38 ± 0.57	7.21 ± 0.24	16.26 ± 0.48	9.87 ± 0.29
8	27.70 ± 0.94	25.82 ± 3.24	60.24 ± 7.57	35.83 ± 0.33	24.05 ± 2.69	80.67 ± 9.03
9	17.24 ± 0.31	29.33 ± 1.65	42.60 ± 2.40	25.67 ± 0.52	28.51 ± 3.12	61.64 ± 6.75
10	24.80 ± 0.21	30.05 ± 0.54	62.77 ± 1.13	36.78 ± 1.64	25.08 ± 2.53	77.69 ± 7.83
11	11.60 ± 0.69	24.97 ± 3.04	24.39 ± 2.97	19.39 ± 0.98	19.40 ± 5.12	31.69 ± 8.37
12	30.76 ± 0.94	26.55 ± 2.33	68.79 ± 6.04	32.30 ± 1.04	16.69 ± 3.07	45.40 ± 8.36
13	19.31 ± 0.59	30.30 ± 2.51	49.28 ± 4.09	29.25 ± 1.76	26.77 ± 2.42	65.95 ± 5.95
14	19.33 ± 5.51	29.99 ± 2.66	48.81 ± 4.33	28.15 ± 2.44	32.77 ± 3.81	77.69 ± 9.02
15	22.24 ± 2.22	31.16 ± 2.69	58.35 ± 5.04	30.37 ± 4.40	28.77 ± 2.18	73.59 ± 5.57
16	20.13 ± 2.22	29.15 ± 1.62	49.41 ± 2.75	30.13 ± 3.32	31.26 ± 2.43	79.30 ± 6.17
17	19.46 ± 2.66	29.55 ± 3.26	48.42 ± 5.34	28.70 ± 2.81	30.13 ± 4.07	72.81 ± 9.84

**Table 5:** Regression analysis of extraction yield and β-glucan content from hot water extraction (HW) and hot alkaline (HA).

Source	Yield-HW (g/100 g)		BG-HW (g/100 g)		Yield-HA (g/100 g)		BG-HA (g/100 g)	
	Sum of	Prob > F	Sum of	Prob > F	Sum of	Prob > F	Sum of	Prob > F
	Squares		Squares		Squares		Squares	
Model	112.84	0.0001	105.75	0.0064	338.48	0.0045	356.88	0.0023
A	36.85	<0.0001	24.20	0.0041	72.14	0.0045	91.8	0.0015
B	42.85	<0.0001	48.28	0.0009	45.44	0.0139	11.27	0.1213
C	0.03	0.8085	1.88	0.2208	5.14	0.3093	11.95	0.1124
A^2^	13.65	0.0009	2.11	0.1179	4.11	0.3595	52.65	0.0066
B^2^	0.30	0.4427	19.69	0.0052	175.04	0.0004	78.16	0.0024
C^2^	2.04	0.0706	0.37	0.9406	13.89	0.1144	62.82	0.0042
AB	10.79	0.0018	3.00	0.1375	0.037	0.9281	5.63	0.253
AC	4.78	0.0139	0.01	0.9102	0.1	0.8802	8.45	0.1708
BC	1.19	0.1482	5.75	0.0584	12.08	0.1366	11.58	0.1172
R^2^	0.9728		0.9372		0.9188		0.9336	
Lack of fit	0.5997		0.1714		0.1105		0.0629	
Adj R^2^	0.9379		0.8564		0.8143		0.8481	
C.V.	7.72		3.84		14.32		7.16	

A = ratio; B = temperature; C = extraction time.

**Table 6:** Regression analysis of extraction yield and β-glucan content from ultrasound-assisted water extraction (UW) and ultrasound-assisted alkaline extraction (UA).

Source	Yield-UW (g/100 g)		BG-UW (g/100 g)		Yield-UA (g/100 g)		BG-UA (g/100 g)	
	Sum of	Prob > F	Sum of	Prob > F	Sum of	Prob > F	Sum of	Prob > F
	Squares		Squares		Squares		Squares	
Model	1116.64	0.0001	174.11	0.0004	1507.44	<0.0001	483.90	0.0008
A	741.13	<0.0001	63.81	<0.0001	1065.02	<0.0001	171.60	0.0002
B	204.84	0.0003	7.94	0.0247	277.36	<0.0001	18.60	0.0547
C	4.69	0.3394	19.19	0.003	50.22	<0.0001	112.70	0.0008
A2	76.43	0.0044	18.10	0.0036	7.33	0.0178	38.20	0.013
B2	11.81	0.1478	0.95	0.3579	7.93	0.015	63.30	0.0038
C2	1.87	0.5380	32.42	0.0007	19.53	0.0015	55.90	0.0052
AB	37.66	0.0228	20.43	0.0026	4.67	0.0435	2.00	0.4778
AC	6.55	0.2650	9.16	0.0183	75.27	<0.0001	3.30	0.3632
BC	33.68	0.0286	0.18	0.6771	0.81	0.3393	0.10	0.8549
R^2^	0.9728		0.9622		0.9964		0.9518	
Lack of fit	0.0687		0.1952		0.5910		0.8853	
Adj R^2^	0.9378		0.9136		0.9918		0.8898	
C.V.	11.38		3.54		3.10		7.49	

A = ratio; B = %amplitude; C = extraction time.

**Table 7:** Validation of optimized extraction conditions for β-glucan yields across different methods.

Conditions	Optimized Process Parameters	Predicted %Yield	Actual %Yield	%Error	Predicted β-Glucan (g/100 g Dry Extract)	Actual β-Glucan (g/100 g Dry Extract)	%Error
HW	1:30, 85 °C, 40 min	13.16	12.94 ± 2.49 ^b^	1.70	30.81	29.74 ± 0.53 ^c^	3.60
HA	1:30, 70 °C, 60 min	12.82	12.59 ± 1.38 ^b^	1.83	33.15	33.10 ± 0.79 ^a^	0.15
UW	1:30, 60% Amplitude, 35 min	37.73	35.16 ± 4.13 ^a^	7.31	35.19	34.51 ± 0.82 ^a^	1.97
UA	1:30, 50% Amplitude, 45 min	35.46	32.97 ± 4.47 ^a^	7.55	33.19	31.99 ± 0.96 ^b^	3.75

Values followed by different letters (a–c) in the same row are significantly different at *p* < 0.05 according to Duncan’s multiple range test.

**Table 8:** Antioxidant and total phenolic effectiveness of each condition.

Conditions	ABTS (µmol TE/g Sample)	DPPH (µmol TE/g Sample)	FRAP (µmol TE/g Sample)	TPC (mg GAE/g Sample)
HW	4.52 ± 0.08 ^b^	3.27 ± 0.19 ^bc^	0.27 ± 0.01 ^bc^	0.21 ± 0.12 ^b^
HA	5.99 ± 0.15 ^a^	3.73 ± 0.02 ^a^	0.42 ± 0.02 ^a^	0.27 ± 0.42 ^a^
UW	3.82 ± 0.02 ^c^	3.12 ± 0.35 ^c^	0.30 ± 0.01 ^b^	0.25 ± 0.17 ^a^
UA	3.12 ± 0.17 ^d^	3.36 ± 0.59 ^b^	0.23 ± 0.01 ^c^	0.26 ± 0.02 ^a^

Values followed by different letters (a–c) in the same row are significantly different at *p* < 0.05 according to Duncan’s multiple range test.

## Data Availability

The original contributions presented in the study are included in the article, further inquiries can be directed to the corresponding author.

## Abbreviations

The following abbreviations are used in this manuscript:

HW	Hot water extraction
HA	Hot alkaline extraction
UW	Ultrasound-assisted water
UA	Ultrasound-assisted alkaline
